# The clinical application progress of multimodal analgesia strategy in enhanced recovery after surgery: a narrative review

**DOI:** 10.3389/fpain.2025.1680157

**Published:** 2025-12-11

**Authors:** Lin Yang, Wang Lou, Yang Jiang, Lina Yang, Dongna Wang, Jiapeng Wang

**Affiliations:** 1 Department of Anesthesiology, Jilin Province FAW General Hospital, Changchun, Jilin, China; 2 Department of Medical Laboratory, Jilin Province FAW General Hospital, Changchun, Jilin, China; 3 Department of Orthopedics, Jilin Province FAW General Hospital, Changchun, Jilin, China

**Keywords:** accelerated rehabilitation surgery, perioperative management, multimodal analgesia, postoperative pain, analgesic drugs

## Abstract

Enhanced recovery after surgery (ERAS) is a multidisciplinary collaborative diagnosis and treatment model based on evidence-based medicine. By optimizing the perioperative management strategy, we can reduce the incidence of postoperative complications, minimize the physiological and psychological trauma stress reaction of patients, shorten the hospitalization period, and promote the functional recovery of patients. The diagnosis and treatment system integrates the advantages of surgery, anesthesiology, nursing, clinical nutrition and other disciplines, and constructs a whole process optimization path through preoperative evaluation, intraoperative management and postoperative rehabilitation, which fully embodies the patient-centered medical service concept. Postoperative pain, as a key factor affecting the rehabilitation process of patients, is closely related to the long-term quality of life of patients. Therefore, the optimization of pain management has become an indispensable and important part of eras. At present, multimodal analgesia (MMA) strategy has been widely recommended as the gold standard for postoperative pain management. This paper aims to review the latest research progress, clinical application strategies and future development direction of MMA in eras. It includes the theoretical basis, core drugs and technologies, application in different surgical fields, impact on patient prognosis, current challenges and future trends of MMA, and provides evidence-based basis for optimizing perioperative pain management.

## 1 Introduction

Enhanced recovery after surgery (ERAS) is based on evidence-based medicine, relying on the cooperation of surgery, anesthesiology, nursing, nutrition and other disciplines, through the optimized clinical path of perioperative treatment, to reduce the physiological and psychological trauma stress of surgical patients, reduce postoperative complications, improve the quality of life of patients and reduce the cost of medical care (1). In 1997, Henrik kehlet (2) and other scholars first proposed the concept of eras, and proposed a multimodal, multi-channel and comprehensive method to reduce the trauma and stress reaction of surgical patients and accelerate the rehabilitation of patients. Eras refers to the selection of various effective methods in the perioperative period, covering more than 20 kinds of intervention measures such as preoperative, intraoperative and postoperative, including the selection of anesthesia methods, multimodal pain relief programs, early nutritional support, avoiding or reducing the use of nasogastric tubes, early postoperative ambulation, resumption of oral feeding, drinking water and other active postoperative rehabilitation measures, in order to reduce the stress response and organ function disorder caused by surgical trauma, maintain the stability of patients' internal environment, reduce postoperative complications, and reduce the cost of hospitalization (3). Therefore, under the new medical model in the 21st century, eras, as a patient-centered surgical quality improvement measure, has promoted surgery and anesthesia to a new level, and has brought significant benefits to patients and the health system (4).

Perioperative pain management is one of the core links in eras clinical pathway. The postoperative acute pain that is not effectively controlled will aggravate the patient's stress response and inhibit the patient's immune function, and 10%–50% of patients will progress to persistent postoperative pain, which will have multiple negative effects on the short-term clinical outcome and long-term quality of life of patients (5). In addition, persistent postoperative pain increases the risk of disability, reduces the quality of life and increases health care expenditure (6). Opioids are the most widely used drugs in traditional postoperative pain management, but they have serious adverse reactions such as respiratory depression, intestinal paralysis, nausea and vomiting (7). Postoperative pain management is closely related to preoperative pain education. Multimodal analgesia (MMA) is a multidisciplinary method that lasts from the early stage before surgery to after surgery admission, and is the key to prevent postoperative patients from acute pain to chronic pain (8). MMA often uses a variety of analgesic drugs and analgesic methods to achieve good analgesic effect, thereby reducing the use of opioids and the adverse reactions caused by opioid use (9). This is to evaluate the clinical value of MMA in eras, analyze the individualized MMA schemes of different surgical types, and explore the direction and challenges of clinical application of MMA in eras in the future.

## 2 Method

A comprehensive literature review was conducted using databases such as PubMed, Cochrane Library, CNKI, and Web of Science in the search strategy. The search covered the period from January 2005 to October 2025 and included both Chinese and English literature. The evaluation was conducted using the PRISMA criteria. The search combined subject terms and free-text terms: subject terms included “perioperative nursing”, “pain management”, and “postoperative complications”; free-text terms included “clinical application”, “efficacy”, “safety”, “pain score”, etc. These terms were connected using “AND” and “OR”. Inclusion criteria: Study types: randomized controlled trials (RCTs), systematic reviews/meta-analyses, and cohort studies. Study subjects: Patients who have undergone surgical procedures, with a sample size of ≥20 cases. Intervention: The experimental group or exposure group explicitly adopted the MMA protocol. Outcome indicators: At least one core indicator should be included, including pain scores (such as VAS/NRS scores), opioid dosage (such as morphine equivalent dose at 24 h/48 h post-surgery), and complication rates (such as nausea and vomiting, respiratory depression, and paralytic ileus). Exclusion criteria: Animal experiments, case reports, review articles (not systematic reviews), and studies with incomplete extractable data. Multiple articles published by the same research team based on the same sample size (only including the latest article with the most complete data).

## 3 ERAS and MMA

### 3.1 Preemptive analgesia

In 1913, American surgeon crile (10) proposed the new analgesic concept of preemptive analgesia, emphasizing the blockade of all harmful stimuli. The idea is to prevent the energy stored in brain cells from being consumed preoperatively, intraoperatively, and postoperatively by preventing the brain from receiving harmful signals or painful stimuli (11). He advocated regional nerve block before general anesthesia for patients to block the transmission of noxious stimuli to the brain during operation, so as to prevent changes in the central nervous system from causing pain and trauma. Woolf (12) later proved its effectiveness through animal experiments, and believed that preventing nociceptive impulses from reaching the center could prevent the occurrence of pain, which was better than treating pain after the occurrence of pain, and proposed the concepts of preemptive analgesia and peripheral sensitization. In other words, preemptive analgesia is to give the patient analgesic drugs in advance before the patient has experienced pain to reduce the physiological response caused by the inward nociceptive signal transmission induced by surgery, so as to reduce or prevent the occurrence of pain (13).

### 3.2 Preventive analgesia

Dionne (14) and other scholars put forward the concept of preventive analgesia, focusing on the implementation quality and duration of analgesic measures, and advocated the use of analgesics before the occurrence of pain, not limited to before surgery, but throughout the whole perioperative process. Compared with conventional treatment, placebo treatment or no treatment, the treatment scheme of preventive analgesia can still observe the phenomenon of pain reduction and analgesic dosage reduction after the duration of intervention (15). Its full name is mainly from skin incision to final complete healing in bed. It prevents central sensitization and completely blocks the transmission of all pain and nociceptive signals from surgical trauma.

### 3.3 Anesthesia and analgesia technology

A wide range of multimodal analgesia include perfect intraoperative anesthesia and analgesia technology, ultrasound-guided nerve block technology, etc. The combined application of different analgesic methods can achieve the maximum analgesic effect, eliminate postoperative pain, strengthen postoperative analgesic effect, and reduce the discomfort of surgical patients.

#### 3.3.1 Local anesthesia or nerve block anesthesia

Anesthetic drugs are injected along the surgical incision or around the surgical area in layers to block the transmission of pain information by nerve trunks and nerve endings, so as to achieve the anesthetic effect. This method can weaken pain signals in the central nervous system and reduce pain signal transmission (16). Both perineural and intravenous dexamethasone and dexmedetomidine are used as adjuncts to local anesthesia to enhance peripheral nerve block properties (17). And the study found that compared with dexmedetomidine, intravenous dexamethasone as the preferred adjuvant to local anesthesia can significantly prolong the duration of sensory and analgesic blockade (18). It has the advantages of little impact on the body, low risk and less postoperative nausea and vomiting reaction, but there may be the risk of local anesthetic overdose or blood poisoning.

#### 3.3.2 Fascial space anesthesia

Fascial space anesthesia is a part of MMA strategy. Compared with other analgesia methods, it is simpler and has various methods, and has good analgesic effect, which can greatly reduce the postoperative adverse reactions of patients. Dalens and other scholars (19) proposed the concept of iliac fascia space anesthesia, which has more reliable analgesic effect than the classic “three in one block”. As a potential space, the iliac fascia space is bounded by the iliopsoas muscle in the rear and the iliac fascia in the front, and the superficial layer of the iliac fascia is covered by the fascia lata. The femoral nerve, obturator nerve, and lateral femoral cutaneous nerve all run close to the back of the iliac fascia at their starting sites, so the iliac fascia lacunar block can block the lateral femoral cutaneous nerve, femoral nerve, and obturator nerve (20). With the development of technology, fascial space anesthesia has the characteristics of simple operation, wide application, reliable curative effect and low complications. It is widely used to reduce the pain of positioning before spinal anesthesia and postoperative analgesia of hip, femur and knee joint in patients with femoral fracture (21).

#### 3.3.3 Intraspinal analgesia

Intraspinal analgesia refers to pushing local anesthetics into the epidural space or subarachnoid space to block spinal nerve roots or spinal cord, cauda equina, etc., so that their corresponding innervation areas produce analgesic effect (22). A prospective, randomized, blinded controlled study found that continuous epidural infusion (CEI) and programmed intermittent epidural bolus (PIEB) have similar analgesic effects for cesarean section pregnant women (23). PIEB with 10 mL of 0.08% ropivacaine and 0.4 μ g/mL sufentanil per hour reduced the incidence of intrapartum fever and had better analgesic effect compared with CEI without any serious maternal and neonatal adverse reactions (24). At delivery, PIEB has outstanding advantages in reducing maternal pain scores and improving maternal satisfaction. However, the optimal volume and dosing interval of PIEB have not yet been determined. The nature and concentration of local anesthetic, the design of epidural catheter and the dosing speed all affect the relevant outcomes of pregnant women and newborns. In future research, PIEB should focus on two dosing modes: 5 mL volume and 30 min frequency (5/30), 10 mL volume and 60 min frequency (10/60) (25).

### 3.4 Postoperative analgesia and MMA

Controlling postoperative pain is the analgesic core of MMA. The MMA strategy emphasizes that the movement pain can be effectively controlled, and the dynamic VAS score is less than 3 points, so as to reduce the postoperative pathophysiological overreaction, promote rehabilitation, and shorten the hospital stay. Patient controlled analgesia (PCA) can be used to treat acute postoperative pain, including intravenous self-control pain, epidural self-control pain and oral drug-controlled pain, among which intravenous self-control pain is considered as the standard of care for postoperative management (26). Compared with the traditional electronic patient-controlled analgesia pump, the infinite analgesia pump system can achieve postoperative pain management more accurately and safely (87). Fentanyl hydrochloride patient-controlled transdermal system is applied to the patient's upper arm or chest, activated by the patient, and uses low-intensity direct current to transfer fentanyl through the skin to the systemic circulation as needed, which can be used for analgesia of moderate to severe pain. Pennington et al. (27) found in two prospective, open label, randomized, phase IIIB clinical trials that in total hip arthroplasty or abdominal or pelvic surgery, patients receiving patient-controlled ion electrophoresis transdermal analgesia with fentanyl hydrochloride were more satisfied with the analgesic effect.

### 3.5 Application status of MMA

In order to achieve optimized pain management, MMA strategy has become a standard scheme in clinical practice, which is the standard scheme in eras. This strategy uses two or more analgesic drugs with different mechanisms of action systematically to target and regulate the multi-level synaptic transmission of peripheral and central nervous system pain transduction pathways, so as to produce a synergistic analgesic effect, further significantly improve the analgesic efficacy, effectively reduce the dosage of opioids, and reduce the risk of related adverse reactions (28). Multimodal analgesia schemes usually integrate a variety of analgesic drugs with different mechanisms of action and regional anesthesia techniques to achieve synergistic effects and optimize pain management (29). Among them, opioid analgesics can mediate analgesia through activating μ receptors in the central nervous system, which is suitable for the control of moderate to severe pain (30). Central COX inhibitors such as acetaminophen have antipyretic and analgesic effects and can reduce the dosage of opioids. Nonselective COX inhibitors simultaneously inhibit COX-1 and COX-2 to exert anti-inflammatory and analgesic effects. Selective COX-2 inhibitors specifically inhibit COX-2 enzymes and reduce gastrointestinal adverse reactions while preserving anti-inflammatory and analgesic effects (31). Alpha-2 agonists (clonidine and dexmedetomidine), anticonvulsants (gabapentin and pregabalin), and local anesthesia techniques through multiple routes during the perioperative period.

At present, for open surgery, due to the large incision and severe pain, opioids + non steroidal anti-inflammatory drugs + intraspinal anesthesia or peripheral nerve block or incision infiltration anesthesia are recommended. Continuous middle thoracic epidural patient-controlled analgesia + non steroidal anti-inflammatory drugs can also control incision pain, but it has the risk of complications such as hypotension, epidural hematoma, urinary retention, etc. it is also recommended that local anesthetic wound infiltration analgesia + abdominal transverse fascia block analgesia + low-dose opioid patient-controlled analgesia + non steroidal anti-inflammatory drugs as an alternative. For laparoscopic surgery, intrathecal or local anesthesia combined with conventional acetaminophen + non steroidal anti-inflammatory drugs is an effective choice (32). For many postoperative pain with unknown causes, such as anastomotic leakage and chronic pain after abdominal surgery, specific problems should be analyzed.

## 4 MMA drug application

In the selection of MMA drugs, the categories covered are extremely extensive, involving non-opioids, opioids, local anesthetics and other categories, and each type of drug includes different subcategories and specific varieties, which have their own characteristics in terms of action targets, analgesic intensity, applicable scenarios and safety, and can meet the analgesic needs of different surgical types, different pain levels and different individual patients (Table 1).

**Table 1:** Different types of MMA drug options.

Drug	Category	Specific drug	Mechanism of action	Main uses	Adverse reactions	References
Non-opioid drugs	Non steroidal anti-inflammatory drugs (NSAIDs)	Parecoxib sodium	A new type of parenteral COX-2 selective inhibitor	Treating mild to moderate pain; assisting in the management of moderate to severe pain and reducing the dosage of opioid drugs; Parecoxib Sodium can be used for short-term postoperative analgesia and reduce the dosage of morphine	Inhibition of COX-1 may increase the risks of postoperative bleeding, gastrointestinal ulcers, and renal insufficiency; it may be associated with anastomotic leakage after gastrointestinal surgery (the evidence is not yet clear); the risks need to be evaluated according to the patient's condition	Amabile and Spencer (2004) (33)
	Non steroidal anti-inflammatory drugs (NSAIDs)	Acetaminophen	It acts on central COX, inhibiting the synthesis and release of prostaglandins and the like; it also inhibits the descending serotonergic pathway and central NO synthesis	It is the first choice for mild to moderate pain; it can reduce the dosage of postoperative opioids by 35%–45%	There is significant hepatotoxicity at therapeutic doses	(Graham et al. (2013) (34)
	α2 receptor agonist	Dexmedetomidine, clonidine	It activates α2 adrenergic receptors, reduces the release of norepinephrine, and produces sedative and analgesic effects	Dexmedetomidine: sedation, analgesia, anti-anxiety, inhibition of stress, reduction of opioid dosage, neuroprotection; Clonidine: enhancement of local anesthetic effect, prolongation of block time	Dexmedetomidine: Low oral bioavailability (16%); Clonidine: Sedation, dry mouth, hypotension, bradycardia (dose-dependent); May prolong recovery time	Nguyen et al. (2017) (35)
	N-methyl-D-aspartate antagonist	Gabapentin (calcium ion modulator)	Down-regulate NR2B, thereby down-regulating TRPV1, pain-related molecules and inflammatory cytokines	Controlling neuropathic pain and specific cancer pain; perioperative analgesia (a single preoperative dose of 600 mg can be used for preemptive analgesia in laparoscopic cholecystectomy)	The evidence for reducing pain scores and opioid dosage with a single application is insufficient; further research on the effects of multiple applications is needed	Aldossari et al. (2025) (36)
Opioid drugs	μ receptor agonist	Sufentanil, fentanyl, hydromorphone	Combining with opioid receptors, activating G protein-coupled receptors, reducing the release of synaptic transmitters, and decreasing neural excitability	Treating moderate to severe acute and chronic pain; Hydromorphone has an analgesic potency 5–10 times that of morphine, takes effect quickly, and can be used for acute pain and as an adjuvant for chronic cancer pain	Nausea, vomiting, pruritus, respiratory depression, potential addiction; There is a problem of drug tolerance	Zöllner et al. (2007) (37)
	κ receptor agonists and μ receptor antagonists/partial agonists	Disosine, nalbuphine, and pentazocin	Activates κ opioid receptors, antagonistic or partially agonizes μ opioid receptors; Dezosin also inhibits norepinephrine and serotonin reuptake	Relieves moderate to severe chronic neuropathic pain and cancer pain; Dezosin can enhance postoperative analgesia in elderly patients, reduce stress response and complication rates	Common opioid adverse reactions (such as nausea, vomiting, etc.)	Liu et al. (2025) (38)
	Opioid receptor antagonists	Naloxone, naltrexone, 6β-naltrexol	Inhibition of opioid receptor-based activity (pure antagonists); Does not affect G-protein receptor basal activity (neutral antagonists)	Adverse effects of antagonists against opioid receptor agonists; 6β-naltrepure does not cause withdrawal reactions; Combination with agonists may reduce drug tolerance	Pure antagonists may cause withdrawal reactions; Long-term use can increase opioid receptor count and pain sensitivity	Inglis et al. (2021) (39)
Local anesthetic medications	Lipid	Bupivacaine	Blocks nerve impulse conduction	Local anesthesia, block anesthesia, and neuraxial anesthesia		Ilfeld et al. (2013) (40)
	Amides	Ropivacaine, lidocaine	Blocks nerve impulse conduction; Ropivacaine has sensory-motor fiber differential blocking properties	Ropivacaine is used for local, block, and neuraxial anesthesia (less cardiotoxic than bupivacaine); Bupivacaine liposomal formulation provides up to 72 h of postoperative analgesia		Casati et al. (2001) (41)

### 4.1 Non opioid drugs

Non opioid drugs mainly include non steroidal anti-inflammatory drugs and paracetamol, which are commonly used for postoperative analgesia in small surgical operations or outpatient operations (42). For the analgesia of patients with moderate to severe pain, such drugs can be used as adjuvant drugs to reduce the application of opioids and reduce adverse reactions. At the same time, producing synergy can improve the analgesic effect, but to avoid the risk, it is not recommended to use two different non steroidal anti-inflammatory analgesic drugs at the same time (43). In addition, highly selective alpha-2 receptor agonists, N-methyl-D-aspartate antagonists, calcium ion modulators, antidepressants, anxiolytics and other drugs play a certain role in perioperative analgesia. Studies have found that oxycodone, as a semi synthetic opioid analgesic, belongs to a highly selective μ-opioid receptor agonist, which can play a role in preventing pain sensitization of tissues around the incision (44).

#### 4.1.1 Non steroidal anti-inflammatory drugs

Non steroidal anti-inflammatory drugs (NSAIDs) are a class of reversible inhibitors of cyclo oxygenase (COX) without steroidal structural basis. They have antipyretic, analgesic, anti-inflammatory and anti rheumatic effects. They are commonly used drugs for the treatment of mild and moderate pain. NSAIDs can effectively inhibit the biological activity of Cox during the metabolism of arachidonic acid in the cell membrane, thereby reducing prostaglandin synthesis, and exert analgesic effects through other peripheral and central mechanisms (45). NSAIDs achieve anti-inflammatory, analgesic and antipyretic effects by inhibiting COX-2, while the increased risk of postoperative bleeding, gastrointestinal ulcers and renal dysfunction is related to the inhibition of COX-1. The serious adverse reactions caused by the use of NSAIDs cause about 400,000 people to be hospitalized every year, with a cost of up to $1.6 billion (46).

Among them, parecoxib sodium, as a new type of parenteral COX-2 selective inhibitor, can be used for the short-term treatment of postoperative pain. Clinical trials in dental, gynecological and orthopedic surgery have shown that parecoxib sodium has good analgesic effect and can save the amount of morphine (33). Studies have found that parecoxib sodium has good safety. It can reduce gastrointestinal side effects (such as nausea, vomiting, constipation and intestinal obstruction) when combined with opioids, does not affect platelet aggregation, and does not cause platelet inhibition when combined with heparin (47). Compared with placebo, perioperative administration of parecoxib sodium can effectively reduce the pain after laparoscopic surgery, and has good safety and no obvious adverse reactions (48). Essex and other scholars (49) found through a large, randomized, double-blind, placebo-controlled trial that parecoxib sodium can relieve postoperative pain of gastrointestinal surgery compared with placebo. Patients receiving parecoxib sodium have less morphine consumption in piglets, and the risk of fatigue and sleepiness of patients is significantly reduced. Previous studies have mostly shown that NSAIDs can accelerate the risk of anastomotic leakage after gastrointestinal surgery (50), however, most studies still have defects, and can not clearly assess whether there is a significant correlation between postoperative NSAIDs and anastomotic leakage, and the use of NSAIDs should be carefully considered in future studies (51). The expert consensus suggested that (52) NSAIDs can be used in pain management until the patient is discharged, but the risk of potential anastomotic leakage, acute kidney injury and other risks should be evaluated according to the patient's age, preoperative coexisting diseases (gastrointestinal diseases, cardiovascular diseases, etc.), operation type, preoperative renal function and other conditions.

Acetaminophen, as one of the most widely used drugs in the world, plays an antipyretic and analgesic role by acting on Cox in the central system, inhibiting the synthesis and release of prostaglandin E1, histamine and bradykinin, and increasing the pain threshold by inhibiting the descending serotonergic pathway and inhibiting central NO synthesis (53). Acetaminophen has weaker analgesic effect than NSAID and COX-2 selective inhibitors, but because of its better tolerance, it is usually the preferred therapeutic drug, but the therapeutic dose of acetaminophen has obvious hepatotoxicity, which should also be carefully selected in clinical treatment (34). The commonly used oral dose for postoperative analgesia is 10–15 mg/kg, which is repeated every 4–6 h, and the maximum daily dose is 50 mg/kg; when combined administration or compound preparation is used, the daily dose is less than 2,000 mg. In addition to being effective for mild and moderate pain, it can reduce the amount of opioids used after surgery by 35%–45% (54).

#### 4.1.2 Alpha-2 receptor agonists

Alpha-2 adrenoceptors are distributed throughout the central and peripheral nervous system, and their main sites of action include the locus coeruleus of pons, spinal cord tracts, ventrolateral medulla oblongata, and spinal dorsal horn. Alpha-2 receptor agonists act on alpha-2 receptors on the presynaptic membrane of noradrenergic neurons, which release less norepinephrine and produce sedation (35). Among them, dexmedetomidine, as a highly selective alpha-2 adrenoceptor agonist, has obvious sedative, analgesic and anxiolytic effects, and was approved by the European Medicines Agency for sedation of patients in the intensive care unit in 2011 (55). Dexmedetomidine can inhibit the discharge of noradrenergic neurons in the locus coeruleus of the brain stem, has a clear central anti sympathetic effect, can effectively inhibit stress, maintain hemodynamic stability, pump before anesthesia induction, realize the easy transition from wakefulness to sleep, induce patients to produce a unique sedative response similar to “natural sleep”, reduce the patient's stress response, and enable patients to cooperate and communicate when stimulated (56). Dexmedetomidine can be administered by intravenous pump injection, intramuscular injection, nasal drip, buccal mucosa or oral administration. However, dexmedetomidine has significant liver first pass elimination effect, and the oral bioavailability is only 16% (57).

At the same time, dexmedetomidine can effectively reduce the perioperative stress response, significantly reduce the perioperative opioid dosage, and prevent postoperative acute pain from turning into chronic pain. Intraoperative use of dexmedetomidine can reduce the occurrence of postoperative shivering, nausea, vomiting, delirium and agitation, especially for patients with hypertension and high intracranial pressure, which can avoid the occurrence of high blood pressure, rapid heart rate and high intracranial pressure when extubating after the withdrawal of narcotic drugs (58). At the same time, animal models and clinical studies have confirmed that intraoperative use of dexmedetomidine has obvious neuroprotective effect, which can improve postoperative cognitive dysfunction in surgical patients after receiving general anesthesia, reduce its incidence, and improve MMSE score (59).

Clonidine combined with local anesthetics has obvious effect on peripheral nerves, and its mechanism of action is relatively complex, which may be related to the mechanism caused by alpha-2 adrenoceptor agonists through promoting C-fiber block, local vasoconstriction, nerve diffusion or axonal retrograde transport, which can enhance the effect of local anesthetics (60). Clonidine injected into nerve sheath alone can not play the role of nerve block. It needs to be combined with other local anesthetics to produce the effect of prolonging the block time through a synergistic mechanism. Its block effect and adverse reactions are dose-dependent (61). A randomized, double-blind controlled trial found that during colonoscopy, the sedation effect of adding clonidine to propofol was better than that of using propofol alone, and propofol demand was less, patient satisfaction was higher, but it may prolong the recovery time of patients (62). Clonidine added to medium or long-acting local anesthetics for peripheral nerve or plexus block can prolong the duration of analgesia and motor block for about 2 h. Common side effects of clonidine include sedation, dry mouth, hypotension and bradycardia, and the higher the dose, the higher the risk of hypotension, fainting and sedation, which may limit its usefulness (63).

#### 4.1.3 N-methyl-D-aspartate antagonists

Gabapentin is a calcium ion regulator. It was first used as an antiepileptic drug and has also been used in perioperative analgesia management in recent years. Gabapentin has been clinically proven to be essential for the control of neuropathic pain in diseases such as diabetic neuropathy and specific types of cancer-related pain in which opioids are ineffective or undesirable (36). In addition, gabapentin treatment can downregulate NR2B, and the downregulation of NR2B expression leads to the downregulation of TRPV1, pain related molecules and inflammatory cytokines, thereby improving peripheral nerve sensitization caused by inflammatory arthritis and alleviating pain symptoms (64). Studies have found that a single preoperative gabapentin 600 mg can be used for effective preemptive analgesia in patients undergoing laparoscopic cholecystectomy (65). However, the evidence of single application of gabapentin to reduce pain scores and reduce opioid dosage is still insufficient. Multiple application of gabapentin before and after surgery may be beneficial to reduce acute and chronic pain, but further mechanistic research is still needed.

### 4.2 Opioids

Opioids are the main drugs for the treatment of acute and chronic pain. They are widely used in clinic and are also the first choice for analgesia. It can reverse bind to opioid receptors and cause conformational changes, activate G protein coupled receptors, and cause related cascades. After the activation of presynaptic opioid receptors, the release of related transmitters is reduced, and the excitability of synaptic nerve cells is reduced (37). Inhibiting the transmission of nociceptive stimuli is the basis of opioid analgesia. Among them, μ receptor agonists are the most widely used opioids with strong analgesic effect. Commonly used drugs include sufentanil, fentanyl, hydromorphone, etc., which are commonly used in the treatment of moderate and severe pain. The potential adverse reactions of opioids, such as nausea, vomiting, skin itching, respiratory depression and potential addiction, make them the main risk factors that trouble clinical application (66).

Hydromorphone is an agonist of μ and δ receptors. Its analgesic intensity is 5–10 times that of morphine. It is widely used in the treatment of acute pain and chronic cancer pain. Its fat solubility is 10 times that of morphine. It is easy to penetrate the blood-brain barrier and act rapidly on the central nervous system (67). Hydromorphone can take effect within 5 min and reach the peak effect within 10–20 min. Because of its obvious peripheral analgesic properties, it can effectively reduce the harmful stimuli in endotracheal intubation, and has obvious peripheral or central mediated analgesic effect. It can be used as an effective pretreatment or adjuvant drug in the process of anesthesia induction (68). In a single center, double-blind, randomized trial, 135 pregnant women with cesarean section were randomly assigned to receive 150 μg intrathecal morphine or 75 μg intrathecal hydromorphone for analgesia. At the dose studied, both intrathecal morphine and intrathecal hydromorphone provided effective analgesia after cesarean section when combined with multimodal analgesia (69).

Agonist antagonist refers to κ receptor agonist and μ receptor antagonist or partial agonist, such as dezocine, nalbuphine, pentazocine, etc. Dezocine, as a leading analgesic in China, is used to relieve moderate to severe chronic neuropathic pain and cancer pain through κ opioid receptor and μ opioid receptor activation as well as norepinephrine reuptake inhibition and serotonin reuptake inhibition (38). A randomized controlled trial exploring the analgesic effect of different doses of dezocine on elderly patients under general anesthesia found that dezocine could effectively enhance the analgesic effect on elderly patients undergoing abdominal surgery under general anesthesia in a dose-dependent manner. In addition, dezocine can significantly reduce the stress response of elderly patients to postoperative tracheal extubation and reduce the incidence of adverse complications after abdominal surgery under general anesthesia (70).

Opioid receptor antagonists are divided into reverse and neutral antagonists, mainly according to their basic activity on opioid receptors. For example, the pure antagonists naloxone and naltrexone can inhibit the basic activity of the receptor. Neutral antagonists such as 6 β—naltrexone are more likely to cause severe withdrawal reactions than others. 6 β—naltrexone does not affect the basic activity of G protein receptors, and has similar antagonistic effects on the adverse reactions of opioid receptor agonists, but does not cause withdrawal reactions (71). Drug tolerance is a common phenomenon after the application of opioids. The combination of opioid receptor antagonists and agonists can reduce drug tolerance, and long-term exposure to opioid receptor antagonists can increase the number of μ, κ, δ receptors and the sensitivity of patients to pain (39).

### 4.3 Local anesthetic drugs

Local anesthetics are divided into lipids and amides. Ropivacaine is a new long-acting amide drug. As a long-acting local anesthetic, it has obvious difference blocking between sensory fibers and motor fibers. Its fat solubility and analgesic efficacy are between lidocaine and bupivacaine, but it has less cardiac toxicity than bupivacaine, so it is preferentially used for local anesthesia, block anesthesia and spinal anesthesia in the perioperative period (41). At present, lidocaine, ropivacaine, bupivacaine and other local anesthetics are commonly used. Bupivacaine is one of the most widely studied and used local anesthetics. It is now provided in the form of liposomal preparation, which is expected to provide up to 72 h of postoperative analgesia when administered as part of peripheral or intraspinal nerve block (40).

## 5 Clinical application of MMA in different surgical fields

### 5.1 Thoracic surgery

Perioperative pain management in thoracic surgery faces many challenges. Local anesthesia infiltration technology and individualized pain management are extremely important. The effectiveness of intravenous acetaminophen in thoracoscopic surgery has been confirmed, and its combination with non steroidal anti-inflammatory drugs or opioid analgesics has a synergistic effect. Among them, regional anesthesia techniques such as thoracic epidural block, paravertebral block and muscle plane block for erector spinae, serratus anterior or intercostal muscles have played the most reliable effect in the postoperative management of cardiothoracic surgery (72). Thoracic epidural anesthesia is considered to be the gold standard for thoracic surgery. It can provide bilateral analgesia by blocking somatic and visceral pain pathways at multiple levels and bilaterally. However, it has high technical requirements for operators. Patients who have bleeding and infection risks at the puncture site, coagulation dysfunction or nervous system disease, and changes in spinal anatomy cannot be operated. In addition, they should be alert to the risks of hypotension, nausea and vomiting, urinary retention, motor block, etc. after operation (73).

The comparison of the efficacy of erector spinae plane block (ESPB) with existing mainstream analgesic techniques (intravenous combined analgesia IV-CA, thoracic epidural analgesia TEA) remains unclear. Bernhard (74) and colleagues conducted a retrospective cohort study in which 61.2% of patients in the thoracoscopic surgery group received ESPB, and 56.9% of patients in the open thoracotomy group received ESPB, showing no significant difference in ESPB application rates between the two groups, providing a basis for comparison. The ESPB group showed a significant reduction in piritramide requirements, with a median dose of 7.5 mg (range 3.0–12.0 mg), significantly lower than the IV-CA group at 10.5 mg (range 6.5–15.5 mg), and the difference was statistically significant (*p* < 0.01). This indicates that in minimally invasive surgeries, ESPB can effectively reduce opioid use and lower the risk of opioid-related adverse effects. However, in open thoracotomy, the ESPB group actually had increased piritramide requirements, with a median dose of 12.0 mg (range 6.0–15.0 mg), significantly higher than the TEA group at 3.0 mg (range 0.0–9.0 mg), and the difference was also statistically significant (*p* < 0.01). This suggests that for more invasive open surgeries, the analgesic strength of ESPB is insufficient to replace TEA, requiring additional opioids. This study clearly delineates the scope of ESPB in thoracic surgery analgesia, with its advantages concentrated in less traumatic minimally invasive procedures, helping reduce opioid dependence and optimize ERAS protocols. In more invasive open thoracotomies, stronger regional analgesic techniques such as TEA are still necessary. This conclusion also provides clinicians with direct evidence-based guidance for developing individualized analgesic plans adapted to the type of surgery, avoiding the blind use of ESPB.

### 5.2 Gastrointestinal surgery

In the field of contemporary surgery, reducing or eliminating the use of opioids is one of the key objectives. Opioids can easily cause gastrointestinal adverse reactions (such as nausea, vomiting, and ileus), fatigue, and may also increase the risk of long-term dependence, which aligns closely with the principles of ERAS (Enhanced Recovery After Surgery) and the long-term quality of life needs of patients. Claes and colleagues (75) conducted a large-scale cohort study involving 842 patients undergoing colorectal surgery to evaluate a care package composed of individualized opioid regimens, routine gabapentinoid administration, and dexmedetomidine as three opioid intervention measures. This was assessed in relation to postoperative opioid consumption in patients undergoing major abdominal surgery (specifically colorectal surgery). During the study period, the proportion of patients with low opioid consumption increased from 35% in 2016 to 66% in 2019, and postoperative opioid use showed a significant declining trend (*P* < 0.001). Among the interventions, routine gabapentin use and individualized opioid regimens were key drivers of the reduction in opioid consumption, while dexmedetomidine, used as a rescue medication, was associated with increased opioid use. Ghadeer and colleagues (76) conducted a prospective cohort study of 344 patients undergoing elective gastrointestinal surgery. They found that 92% of patients used acetaminophen and 38% used nonsteroidal anti-inflammatory drugs (NSAIDs), reflecting the basic framework of multimodal analgesia (MMA). Additionally, 67% of patients received a transverse abdominis plane block, indicating that perioperative non-opioid analgesic techniques are being emphasized, although high opioid prescription rates at discharge have not been completely eliminated. This study challenges the traditional notion that opioids must be used postoperatively and suggests that prescription plans should be stratified based on individual patient characteristics such as age, preoperative anxiety, and type of surgery. For high-risk patients, such as younger individuals with high preoperative anxiety undergoing rectal resection, opioid prescriptions may be retained but at strictly controlled doses. Conversely, for low-risk patients, non-opioid medications combined with regional anesthesia should be prioritized, implementing an opioid-free strategy to avoid over-prescription.

Devon and other scholars (77) conducted a retrospective cohort study including 627 patients who underwent anorectal surgery. The study found that both the prescription rate and dosage of opioids were clearly stratified according to the degree of surgical trauma, with the highest prescription rate for sacrococcygeal abscess excision (96%), followed by hemorrhoidectomy (87%), fistula surgery (78%), incision and drainage (71%), and the lowest prescription rate for examinations under anesthesia (49%). The study suggested that the feasibility of low-opioid or opioid-free analgesia in anorectal surgery should be further validated, recommending that clinicians prioritize combining non-opioid drugs (such as acetaminophen and nonsteroidal anti-inflammatory drugs) with local analgesia (such as wound infiltration anesthesia). However, for most patients, regardless of the type of anorectal surgery performed, pain has little impact on daily activities, and postoperatively, patients can achieve adequate pain management with no more than 5–10 doses of 5 mg oxycodone.

### 5.3 Orthopedic surgery

MMA pathway can promote the rehabilitation of patients by shortening the length of hospital stay, reducing postoperative complications, and improving the prognosis and satisfaction of patients, showing great advantages in the anesthesia and postoperative management of orthopedic patients. Compared with general anesthesia and intraspinal block in patients undergoing outpatient orthopedic surgery, the use of peripheral nerve block for anesthesia can significantly shorten the length of hospital stay of patients (78). Choi and other scholars (79) summarized and determined the MMA scheme in orthopedic surgery, including pre admission consultation and optimization of medical conditions during admission. Before operation, the methods of avoiding long-term fasting, multimodal analgesia and preventing postoperative nausea and vomiting were determined. During the operation, the anesthesia program, prevention of hypothermia and fluid management, catheterization, antibacterial prevention, blood preservation, local infiltration analgesia and local nerve block, and surgical factors were determined. Early oral nutrition, thromboembolism prevention, early activities and discharge plan were determined after operation. MMA in orthopedic surgery reduces postoperative complications, hospital stay and costs, and improves patient prognosis and satisfaction with accelerated rehabilitation.

Scholars such as Sachiyuki (80) conducted a prospective, single-center, randomized controlled trial to evaluate the effects of postoperative epidural analgesia and intraoperative periarticular injection on pain control after total knee arthroplasty under spinal anesthesia. The study found that the postoperative pain control score for periarticular injection was 788, significantly better than the score of 1,065.9 in the epidural analgesia group, with the difference being statistically significant (*p* = 0.0059). At the same time, periarticular injection could better relieve postoperative pain, allow earlier recovery of knee flexion angle, and reduce the incidence of nausea. However, when using periarticular injection, attention should be paid to avoid transient peroneal nerve palsy. Zhang and colleagues (81), through a clinical randomized controlled trial, compared the effects of combined lumbar plexus and sciatic nerve block with combined spinal and epidural anesthesia on anesthesia and postoperative analgesia in patients with unilateral lower limb fractures. The study found that for patients undergoing internal fixation for unilateral lower limb fractures, multiple injections via lumbar plexus or sciatic nerve catheters had analgesic effects comparable to continuous epidural analgesia, but multiple injections via lumbar plexus or sciatic nerve catheters had three significant advantages. First, they provided better hemodynamic stability, avoiding hypotension after anesthesia and in the early postoperative period. They also reduced the risk of complications, with a lower incidence of urinary retention, promoted faster recovery of gastrointestinal function, shortened the time to first oral intake, and had minimal adverse reactions, resulting in superior overall clinical outcomes.

### 5.4 Endoscopic surgery

MMA plays an important role in promoting postoperative recovery. Yang and other scholars (82) conducted a prospective, randomized, controlled clinical trial. 120 adult patients undergoing laparoscopic gastrointestinal surgery were randomly assigned in a ratio of 1:1:1:1. The patients received one of the patient-controlled analgesia regimens (oxycodone or sufentanil) and one of the two regional blocks (quadratus psoas block or transvs. abdominis plane block). The postoperative recovery quality of patients undergoing eras laparoscopic gastrointestinal surgery was greatly improved. And MMA can effectively prevent postoperative gastrointestinal dysfunction in patients undergoing laparoscopic colorectal surgery and accelerate the rehabilitation process of patients (83).

Non-opioid drugs, as part of MMA, have certain advantages in managing acute postoperative pain. Omer and colleagues (84) conducted a prospective, randomized, double-blind study comparing the analgesic effects of pregabalin monotherapy and pregabalin combined with intravenous ibuprofen, two non-opioid analgesic regimens, after laparoscopic cholecystectomy. The study found that a preoperative analgesic regimen of 150 mg pregabalin combined with 400 mg intravenous ibuprofen resulted in lower pain scores throughout the postoperative period (especially during movement), providing more comprehensive analgesia. This combination analgesia regimen reduced postoperative fentanyl consumption by 55%, and 14% of patients did not require any opioids, significantly improving the issue of opioid dependence.

### 5.5 Cesarean section

Post-cesarean section pain combines both somatic pain (abdominal wall incision pain) and visceral pain (uterine contraction pain), requiring regional block techniques that address both types of pain to meet the needs of MMA. At present, cesarean section surgery lacks the gold standard of peripheral regional analgesia.

The quadratus lumborum block (QLB), as a well-established technique, has been proven to effectively cover the lower abdominal analgesic area. Although the erector spinae plane block (ESPB) has been applied in other lower abdominal surgeries, previous studies have mostly focused on the T9 level. There is still a lack of research on the analgesic effect of T12-level ESPB after cesarean section and its comparison with bilateral L2–L3 transmuscular QLB (TQLB). There is no gold standard for peripheral regional analgesia in cesarean section. Reesha and colleagues conducted a prospective, randomized, noninferiority trial comparing the postoperative analgesic effects of ultrasound-guided erector spinae plane block and transmuscular quadratus lumborum block after cesarean section. The study found that both provided comparable analgesia and could be part of MMA for cesarean patients. Thomas and colleagues conducted a prospective, randomized, blinded, controlled study to verify whether bilateral T12-level ESPB is non-inferior to TQLB for postoperative analgesia after cesarean section. There were no significant differences between the two groups in total tramadol consumption within 48 hours postoperatively, time to first rescue analgesia, overall patient satisfaction, or safety. Additionally, the ESPB group showed superior control of resting pain at 6 hours postoperatively and movement pain at 4–6 hours and 36 hours, indicating certain advantages (89).

The study believed that intrathecal morphine injection could be considered as an alternative method for cesarean section patients who were intolerant to morphine. The combined use of tramadol and flurbiprofen can produce antagonism and enhance the analgesic effect after cesarean section (85). It should be noted that 80% of cesarean section and intraspinal anesthesia parturients are prone to intraoperative pain and postoperative nausea, vomiting and hypotension. Optimizing anesthesia strategy and surgical management department can reduce the use of opioids and further reduce the risk of postoperative nausea and vomiting (86). Pain management is crucial for achieving better clinical outcomes and postoperative recovery. With the continuous development of drugs, there will be more and better drug treatment options available in the future. To facilitate the presentation of the clinical application of MMA strategy in different surgical fields, relevant information is summarized in Table 2.

**Table 2:** Summary of clinical research on MMA in different surgical fields.

Surgical category	Research type	Sample size	Intervention	Outcome measure	Core conclusion	References
Thoracic surgery	Retrospective cohort study	There were 165 VATS patients. 72 patients underwent open chest surgery	The experimental group in VATS was given ESPB, while the control group was given IV-CA; During open chest surgery, the experimental group was given ESPB, while the control group was given TEA	The dosage of piracetam used, patient pain score, and incidence of analgesic related adverse events	ESPB can serve as an effective supplement to IV-CA after VATS surgery, reducing the use of opioid drugs and achieving comparable analgesic effects. After open chest surgery, TEA remains a superior analgesic option, with better efficacy in reducing opioid use than ESPB	Zapletal et al. (2025) (74)
Gastrointestinal surgery	Retrospective cohort study	842 patients underwent major colorectal surgery	Evaluate the association between a nursing package consisting of individualized opioid regimens, conventional gabapentin, and clonidine as rescue analgesics, and postoperative opioid consumption in patients undergoing major abdominal surgery	The amount of intravenous and oral opioid drugs administered in the hospital on the day of surgery and the first 5 days after surgery	The combination of gabapentin and personalized opioid regimens is a key factor driving the decrease in opioid use, while clonidine, as a salvage medication, is associated with an increase in opioid use	Gedda et al. (2023) (75)
	Prospective cohort study	344 patients who underwent major colorectal surgery	The study only collected perioperative nursing characteristics of patients, including pain relief prescriptions at discharge (such as acetaminophen, nonsteroidal anti-inflammatory drugs, opioid drugs), and did not actively apply new intervention plans	Comparison of prescription and consumption of opioid drugs at discharge after elective colorectal surgery	The prescription of opioid drugs at discharge after colorectal surgery is significantly higher than the actual consumption, and there are clear predictive factors for consumption	Olleik et al. (2025) (76)
	A mixed design of retrospective cohort study combined with prospective cross-sectional survey	627 patients undergoing outpatient anorectal surgery	Observing the situation of doctors prescribing opioid drugs after anal and rectal surgery in different outpatient clinics in routine clinical practice, as well as the actual use and pain management effects of patients, without actively intervening in prescription or medication behavior	The prescription amount of opioid drugs at discharge, the supplement status of opioid drug prescriptions, and the actual usage reported by the patient. By using validated assessment tools, measure the intensity of postoperative pain and the degree of interference of pain on daily activities in patients, and determine whether pain management is sufficient	There is a phenomenon of excessive prescription of opioid drugs after outpatient anorectal surgery, and most patients can meet their pain management needs with low-dose medication	Livingston-Rosanoff et al. (2020) (77)
Orthopedic surgery	Prospective, single center, randomized controlled trial	111 patients undergoing unilateral total knee arthroplasty.	Experimental group: Postoperative analgesia was administered by injecting periarticular analgesics. Control group: Postoperative analgesia was administered using epidural analgesia.	The primary indicator is postoperative resting pain, quantified by the area under the Visual Analog Pain Scale (VAS) score curve at 72 h after surgery. Secondary indicators include knee flexion angle on postoperative day 1 and day 2, as well as incidence of adverse reactions such as postoperative nausea and transient peroneal nerve paralysis	Periarticular injection is superior to epidural analgesia in terms of postoperative analgesic effect and partial recovery indicators, but specific adverse reactions should be noted	Tsukada et al. (2014) (80)
	Randomized controlled trial	70 patients planning to undergo internal fixation surgery for unilateral lower limb fractures	N group (peripheral nerve conduit group): 35 cases E group (continuous epidural analgesia group): 35 cases	Surrounding the postoperative analgesic effect, adverse reactions, and recovery related indicators: Analgesic effect: The degree of postoperative pain is measured by visual analog scale (VAS) at different time points. Other indicators: the mean arterial pressure 30 min after anesthesia and 4 h after operation, the incidence of urinary retention, and the time when the patient first took food	The pain relief effects of the two analgesic methods are comparable, but the safety and recovery convenience of multiple injections through peripheral nerve conduits are better	Zhang et al. (2013) (81)
Endoscopic surgery	Prospective, randomized, controlled clinical trial using a 2 × 2 factor design	120 adult patients underwent laparoscopic gastrointestinal surgery	Combination 1: Patient controlled analgesia based on hydrocodone + lumbar muscle block (QLB) Combination 2: Patient controlled analgesia based on hydrocodone + transverse abdominis plane block (TAPB) Combination 3: Patient controlled analgesia based on sufentanil + lumbar muscle block (QLB) Combination 4: Patient controlled analgesia based on sufentanil + transversus abdominis plane block (TAPB)	Quality of recovery 24 h after surgery: Quantitatively evaluate the quality of recovery of patients 24 h after surgery using the 15 item Quality of Recovery Scale (QoR-15)	MMA strategy has great potential in postoperative analgesia	Yang et al. (2024) (82)
	Prospective randomized controlled study	108 patients undergoing elective laparoscopic colorectal surgery	Experimental group: Flurbiprofen, Acertide, and Oxycodone were administered before skin incision, and bilateral transversus abdominis plane block (TAPB) was performed before anesthesia induction. Control group: Administered sufentanil, flurbiprofen aceti, and bilateral TAPB in the post anesthesia intensive care unit (PACU)	Postoperative gastrointestinal function: incidence of postoperative gastrointestinal dysfunction (POGD), I-FEED score (a scale used to evaluate postoperative gastrointestinal function). Other indicators: postoperative inflammatory factor levels (lipopolysaccharide LPS, C-reactive protein CRP, tumor necrosis factor TNF—α, interleukin-6 IL-6), rehabilitation indicators, postoperative pain assessment, organ complications	MMA strategy can effectively improve postoperative gastrointestinal function and inflammatory status in patients undergoing laparoscopic colorectal surgery	Liu et al. (2021) (58)
	Prospective, randomized, double-blind controlled study	58 patients who underwent laparoscopic cholecystectomy	Control group: Only 150 mg Pregabalin was given before surgery. Experimental group: 150 mg pregabalin and 400 mg intravenous ibuprofen were administered before surgery. Both groups received patient-controlled intravenous injection of fentanyl for postoperative analgesia	Centered around the postoperative analgesic effect and opioid consumption, the core indicators include: Pain score: Visual Analog Scale (VAS) for postoperative PACU rest for 1 and 2 h, as well as exercise for 1, 2, 4, 12, and 24 h. Opioid related: total postoperative fentanyl consumption, demand for additional rescue analgesics, and number of users. Recovery indicator: Post anesthesia care unit (PACU) stay time	The preoperative analgesic regimen of pregabalin combined with ibuprofen has a better analgesic effect and can significantly reduce postoperative opioid use	Karaca et al. (2019) (84)
Cesarean section	Prospective, randomized, non inferiority trial	124 patients undergoing cesarean section	Both groups were injected with 20 mL of 0.25% ropivacaine on each side during block, and received 2-day prophylactic treatment with acetaminophen and ketorolac. ESPB group: bilateral erector spinae plane block (ESPB) was performed at the T12 level. TQLB group: Bilateral transmuscular lumbar block (TQLB) was performed at the L2-L3 level	The primary indicator is the total consumption of tramadol within the first 48 h after surgery. Secondary indicators: Cumulative tramadol consumption at different time points, postoperative resting and exercise numerical rating scale (NRS) scores, time to first rescue analgesia, incidence of block related complications, and patient satisfaction with analgesia	The analgesic effect of T12 level bilateral ESPB after cesarean section is not inferior to that of L2–L3 level bilateral TQLB	Joshi et al. (2024) (88)
	Prospective, randomized, blinded, controlled study	104 patients undergoing cesarean section	Posterior lumbar muscle block group: During cesarean section under spinal anesthesia, bilateral posterior lumbar muscle block is performed. Intrathecal injection of morphine group: During cesarean section under spinal anesthesia, intrathecal injection of morphine is performed	The primary indicator is the cumulative use of morphine administered intravenously by the patient within 24 h after surgery. Secondary indicators: Accumulated morphine use within 48 h after surgery, static/dynamic pain score (NRS), functional recovery status (ObsQoR-11 questionnaire), and incidence of adverse reactions (such as itching)	The analgesic effect of posterior lumbar muscle block is comparable to that of intrathecal injection of morphine, and it has advantages in safety and functional recovery	Giral et al. (2024) (89)
	Retrospective analysis	2,323 patients undergoing cesarean section	Three postoperative analgesia regimens based on tramadol: Group T: Use tramadol alone for analgesia. TF group: Use a mixture of tramadol and flurbiprofen aceti for analgesia. TB group: Use a combination of tramadol and butorphanol for analgesia	Analgesic effect: The incidence of “insufficient analgesia” in resting pain and exercise pain within the first 48 h after surgery (defined as a numerical rating scale NRS score ≥4). Postoperative recovery: percentage of out of bed activity 2 days after surgery, and recovery rate of intestinal function 2 days after surgery	There are significant differences in the analgesic effect and recovery impact of different combination regimens of tramadol, and the combination of tramadol and flurbiprofen aceti is the most optimal	Yang et al. (2022) (85)

## 6 Discussion

Under the guidance of the ERAS concept, MMA has progressed beyond the initial stage of merely combining various analgesic methods and has entered a new level of synergistic interventions based on pain mechanisms. Perioperative pain is essentially a multidimensional pathological process involving peripheral nociceptive stimuli, central sensitization, and psychological cognitive amplification. Traditional single-modal analgesia (such as using opioids alone) can only block one link in this process, often resulting in insufficient pain relief or over-reliance on opioids. The core logic of modern MMA is to achieve mechanistic complementarity through non-opioid drugs (such as acetaminophen, nonsteroidal anti-inflammatory drugs), regional blockade techniques (such as fascial plane blocks, nerve plexus blocks), and psychological interventions (such as preoperative anxiety counseling), targeting three key links: peripheral inflammatory response, neural signal transmission, and central pain integration, forming a synergistic analgesic effect where 1 + 1 > 2. Their combination can significantly reduce opioid consumption, avoiding opioid-related adverse effects (such as gastrointestinal motility suppression and respiratory depression) while also lowering the risk of postoperative chronic pain caused by central sensitization. This aligns closely with the core goals of ERAS in reducing surgical stress and promoting rapid recovery of organ function.

It should be emphasized that the implementation of preventive MMA must be based on the individualized assessment of patients, comprehensively considering the objective factors such as disease characteristics, diagnosis and treatment processes, and resource allocation of medical institutions, so as to avoid the mechanized understanding of eras concept. This process requires the establishment of a multidisciplinary collaboration mechanism, especially the integration of professional support from surgery, nursing, rehabilitation medicine and psychology.

The application of MMA in eras needs to implement the concept of precision analgesia and individualized pain management. At present, the rapid development of artificial intelligence and computer technology has brought new opportunities for analgesic management. The postoperative patient-controlled analgesia system based on the information platform can significantly optimize pain interventions, improve the quality of analgesia, achieve the goal of precision medicine, and promote the clinical practice of comfort medicine by intelligently transforming the wireless analgesia management system. The deepening of pharmacological research and the iterative updating of target controlled infusion (TCI) equipment have promoted the progress of clinical application of tci-pca technology. As a good scheme of patient-controlled analgesia, tci-pca can well overcome the problem of opioid concentration fluctuation. The development of ultrasound technology has pushed multimodal analgesia to a new level, such as fascial space block, nerve stem, nerve root and plexus block, which are increasingly used for precision anesthesia and postoperative analgesia. For example, iliac fascia space block has a good preoperative and postoperative analgesic effect for hip and femoral surgery. With the advent of ultra long acting local anesthetics, perioperative regional nerve block technology has opened up a new era for postoperative pain management.

## 7 Conclusion

Under the guidance of the ERAS concept, MMA has achieved a phased leap from the simple superposition of various analgesic methods to mechanism-based synergistic intervention, and has become the core strategy for perioperative pain management. The multidimensional pathological characteristics of perioperative pain determine the limitations of unimodal analgesia. In contrast, modern MMA forms a synergistic effect targeting key links such as peripheral inflammation, nerve signal transmission, and central pain integration through the organic combination of non-opioid drugs, regional block techniques, and psychological intervention. This not only significantly reduces opioid consumption and related adverse reactions, but also decreases the risk of postoperative chronic pain, which is highly consistent with the core goals of ERAS, namely reducing surgical stress and promoting the rapid recovery of organ functions. However, the standardized implementation of MMA needs to be based on individualized patient assessment, comprehensively considering objective factors such as disease conditions, diagnosis and treatment processes, and medical resources. At the same time, it relies on the support of a multidisciplinary collaboration mechanism involving surgery, nursing, rehabilitation medicine, and psychology, and runs through the core concepts of precise analgesia and individualized management.
